# Investigating the cecal microbiota of broilers raised in extensive and intensive production systems

**DOI:** 10.1128/spectrum.02352-23

**Published:** 2023-09-27

**Authors:** Camila S. Marcolla, Tingting Ju, Hannah L. Lantz, Benjamin P. Willing

**Affiliations:** 1 Department of Agricultural, Food and Nutritional Science, University of Alberta, Edmonton, Canada; University of Minnesota Twin Cities, St. Paul, Minnesota, USA

**Keywords:** broiler, gut microbiota, cecal microbiota, poultry, extensive production system, intensive production system, commensal

## Abstract

Intensive broiler production practices are structured to prevent the introduction and spread of pathogens; however, they can potentially minimize the exposure of broilers to beneficial commensal bacteria. In this study, we used 16S rRNA amplicon sequencing to characterize the cecal microbiota of 35-day-old broilers from 22 independent commercial farms rearing broilers under intensive (IPS) or extensive production systems (EPS). We aimed to determine which bacteria are normal inhabitants of the broiler ceca and which bacteria might be missing from broilers in IPS. In addition, we generated a collection of 410 bacterial isolates, including 87 different species, to be used as a resource to further explore the effects of selected isolates on bird physiology and to elucidate the role of individual species within the cecal microbial community. Our results indicated significant differences in the microbiota of broilers between systems: the microbiota of broilers from EPS was dominated by Bacteroidetes {55.2% ± 8.9 [mean ± standard deviation (SD)]}, whereas Firmicutes dominated the microbiota of broilers from IPS (61.7% ± 14.4, mean ± SD). Bacterial taxa found to be core in the EPS microbiota, including *Olsenella*, *Alistipes*, *Bacteroides*, *Barnesiella*, *Parabacteroides*, *Megamonas*, and *Parasutterella*, were shown to be infrequent or absent from the IPS microbiota, and the EPS microbiota presented higher phylogenetic diversity and greater predicted functional potential than that of broilers in IPS. The bacteria shown to be depleted in broilers from IPS should be further investigated for their effects on bird physiology and potential application as next-generation probiotics.

**IMPORTANCE:**

Production practices in intensive farming systems significantly reduce the introduction and spread of pathogens; however, they may potentially minimize the exposure of animals to beneficial commensal microorganisms. In this study, we identified core bacteria from the cecal microbiota of broilers raised in extensive production systems that are missing or reduced in birds from intensive systems, including *Olsenella*, *Alistipes*, *Bacteroides*, *Barnesiella*, *Parabacteroides*, *Megamonas*, and *Parasutterella*. Furthermore, the cecal microbiota of broilers from extensive systems showed higher diversity and greater functional potential than that of broilers from intensive systems. In addition, a collection of bacterial isolates containing 87 different species was generated from the current study, and this important resource can be used to further explore the role of selected commensal bacteria on the microbial community and bird physiology.

## INTRODUCTION

The community of bacteria living in the gastrointestinal tract (GIT) of poultry has been shown to influence many aspects of host physiology, including nutrient digestion (1 – 3), immune system maturation and tuning (4 – 6), disease resistance and tolerance (7 – 9), and intestinal development (10). Throughout evolution, this association has resulted in bacteria that are highly adapted to the GIT of specific host species (11), that are likely to colonize the GIT after a single exposure (12), and that can significantly affect host metabolism (13). Moreover, the microbiota can respond quickly to environmental conditions and promote changes in host phenotype that aid acclimation and adaptation to new situations (14, 15). Therefore, it is expected that individuals hosting beneficial bacteria are more likely to survive, reproduce, and transmit their commensal bacteria to the next generation, favoring the persistence of both the host species and beneficial commensal organisms (15, 16).

Despite growing evidence indicating the importance of proper microbial colonization (17), intensive farming practices are structured in a way that reduces opportunities for the selection and transmission of beneficial commensal bacteria across generations. In intensive production systems (IPS), broilers are hatched in artificial hatcheries and moved to enclosed barns, which limit contact with the microbiota of mature birds. In addition, broilers in IPS are fed standardized diets with a limited number of ingredients, which may contain antibiotics. In contrast, broilers reared in extensive production systems (EPS) might have access to outdoor environments and be fed antibiotic-free diets, which offer more opportunity for exposure and colonization by a wide variety of microorganisms. In face of the restrictions on the use of antibiotics due to concerns about the development of antimicrobial resistance, there is a need to find alternative strategies to promote animal growth and control disease occurrence in IPS. The restoration of the chicken’s native microbiome using next-generation probiotics, which include bacterial strains that reside in the intestinal ecosystem, could be a potential alternative (18). This contrasts with “traditional” probiotic products that usually contain bacterial strains harvested from soil and fermented food, which may fail to effectively colonize and persist in the chicken GIT unless constantly provided (12, 19). The lack of host-adaptation traits coupled with individual variabilities in broiler microbiota communities could be the reason why probiotics that aim to improve performance and disease resistance have inconsistent results (20, 21).

Domesticated animals colonized with microbiota from wild counterparts have shown reduced inflammatory responses and increased survival following infection challenges (22), and their microbiota was shown to be more resilient to disturbances caused by factors such as dietary and environmental changes as well as antibiotic use (23). Bacteria from chickens from EPS were shown to have higher antagonist activity against pathogens and less resistance to antimicrobials than bacteria from chickens from IPS (24, 25). In addition, studies have shown that poultry raised in IPS may lack the core bacteria that are found in free-range and feral birds (26 – 28).

Previous studies comparing the microbiota of poultry raised in IPS and EPS presented some limitations, such as small sample sizes and the unavailability of samples from similar-aged birds from each system (25, 28). In the present study, we characterized the cecal microbiota of age-matched broilers from 22 independent commercial farms under IPS (*n* = 12 farms) or EPS (*n* = 10 farms), aiming to determine which bacteria are normal inhabitants of the chicken GIT and which bacteria might be missing from broilers in IPS. Further genomic characterization of selected commensal bacterial isolates has also been performed to guide the development of next-generation probiotics and further studies aiming to understand the role of individual bacteria within the gut microbiota.

## RESULTS

The amplicon sequencing of 105 cecal samples generated an average of 52,457 ± 36,444 [mean ± standard deviation (SD)] reads that were assigned to 12,331 amplicon sequence variants (ASVs) and 792 taxa. Samples were rarefied at 11,360 reads, resulting in the removal of 4,172 ASVs and 100 taxa that were not present in any sample after random subsampling. Two samples that presented less than 11,360 reads were excluded from downstream analysis. Analyses of 103 rarefied samples (*n* = 45 from EPS and *n* = 58 from IPS) indicated the presence of 8,159 ASVs and 692 taxa.

### Production systems significantly affect cecal microbiota composition

**FIG 1:**
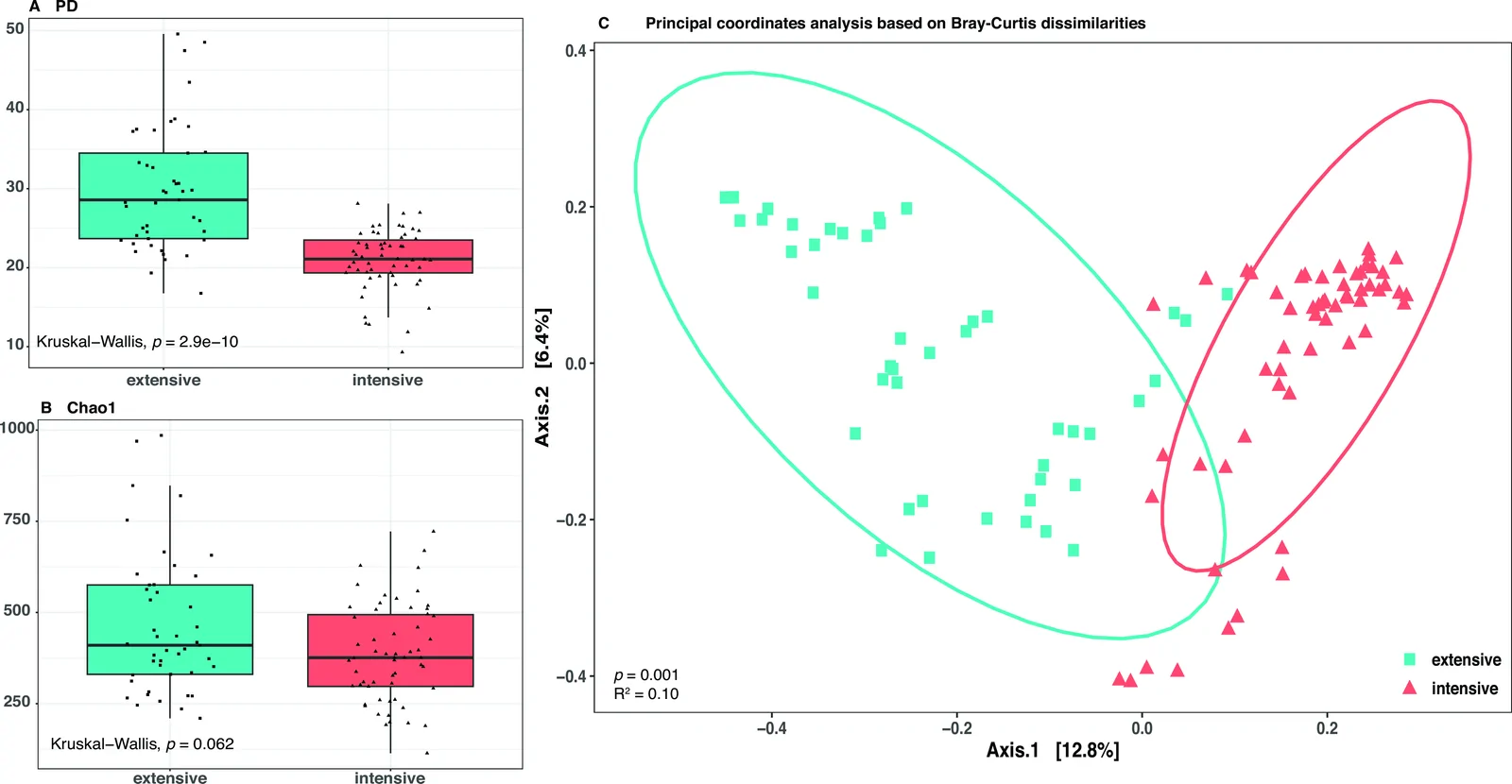
(**A, B**) Alpha-diversity indices and (**C**) PCoA generated based on Bray-Curtis dissimilarity of cecal samples obtained from 35-day-old broilers from extensive or intensive production systems. Samples are colored and shaped according to treatments, and data ellipses represent the 95% confidence region for group clusters assuming a multivariate *t*-distribution.

**FIG 2:**
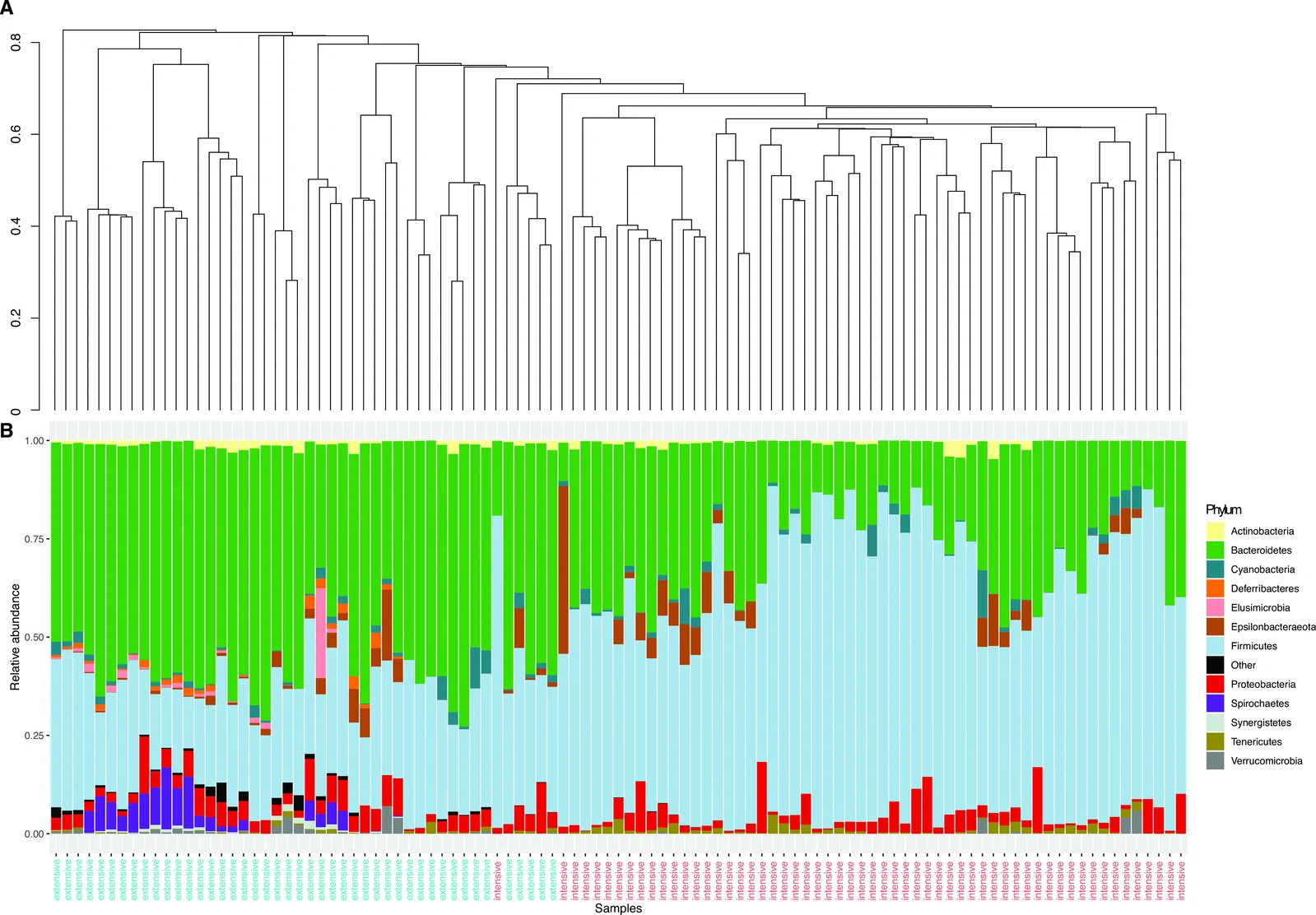
(**A**) Dendrogram showing hierarchical clustering and (**B**) bar plots showing the relative abundance of phyla in cecal samples obtained from 35-day-old broilers from extensive or intensive production systems. Phyla observed in less than 5% of samples and that had less than 1% relative abundance were combined as “other” (black).

The cecal microbiota of broilers raised in EPS had higher phylogenetic diversity than that of birds in IPS (*P* < 0.001) (Fig. 1A), but no significant differences were observed in alpha-diversity indices including Chao1 (Fig. 1B), Shannon, and Simpson (Fig. S1) (*P* = 0.06, *P* = 0.93, and *P* = 0.35, respectively). Permutational multivariate analysis of variance (PERMANOVA) analysis indicated that the microbiota composition was significantly different between systems (*P* = 0.001, *R* ^2^ = 0.10) (Fig. 1C). Hierarchical clustering analysis showed a clear separation of samples according to rearing system, except for a single sample obtained from a broiler from IPS that was clustered with samples obtained from broilers from EPS (Fig. 2A). In addition, samples of birds from the same farm clustered tightly, except for three samples from three different farms that were separated from the remaining samples obtained from the same farm (Fig. S2). Bacteroidetes dominated the cecal microbial community in EPS birds [55.2% ± 8.9 (mean relative abundance ± SD)], whereas Firmicutes dominated the cecal microbial community in IPS broilers (61.7% ± 14.4) (Fig. 2B). Six phyla were exclusively detected in broilers from EPS, including Deferribacteres (0.8% ± 1.1, *P* < 0.001), Elusimicrobia (0.9% ± 3.4, *P* < 0.001), Fusobacteria (0.1% ± 1.3, *P* < 0.001), Patescibacteria (0.3% ± 0.8, *P* < 0.001), Spirochaetes (2.7% ± 4.1, *P* < 0.001), and Synergistetes (0.4% ± 0.5, *P* < 0.001). Besides the six unique phyla, the cecal microbiota of EPS broilers presented a higher relative abundance of Actinobacteria (1.2% ± 0.9 vs 0.7% ± 1.1, *P* < 0.001), Bacteroidetes (55.2% ± 8.9 vs 27.9% ± 11.9, *P* < 0.001), *Lentisphaera* (0.5% ± 0.5 vs 0.01% ± 0.04, *P* < 0.001), Proteobacteria (4.7% ± 3.0 vs 0.3% ± 1.0, *P* = 0.002), and Verrucomicrobia (0.7% ± 1.3 vs 0.3 ± 1.0, *P* < 0.001) than that of IPS broilers. On the other hand, IPS broilers had enriched Firmicutes (61.7% ± 14.4 vs 28.7% ± 7.5, *P* < 0.001) and Tenericutes (1.0% ± 1.1 vs 0.4% ± 0.6, *P* = 0.003). The most abundant phyla within the microbiota of broilers in both systems were Firmicutes, Bacteroidetes, and Proteobacteria (Fig. S3).

### EPS cecal microbiotas harbor unique ASVs

Among the total number of ASVs detected, 52.5% were found to be unique to birds raised in EPS, 33.4% were unique to birds raised in IPS, and 14.3% were shared between both systems. An average of 67.8% ± 14.4 (mean relative abundance ± SD) of the cecal microbial community in EPS broilers was composed of ASVs that were unique to this system, whereas the ASVs unique to the microbiota of IPS broilers composed only 14.5% ± 6.7 of the cecal microbial community in IPS broilers (Fig. S4A). We further looked at phyla assignment for shared and unique ASVs and found that Bacteroidetes was the most abundant phyla detected within the unique microbial members of extensively and intensively raised broilers, composing 71.2% ± 10.9 of the unique community of EPS and 46.6% ± 22.9 of the unique community in IPS (Fig. S4B). Most of the ASVs shared by both systems were Firmicutes, with relative abundances of 73.7% ± 17.4 in EPS and 72.5% ± 15.0 in IPS (Fig. S4C).

**FIG 3:**
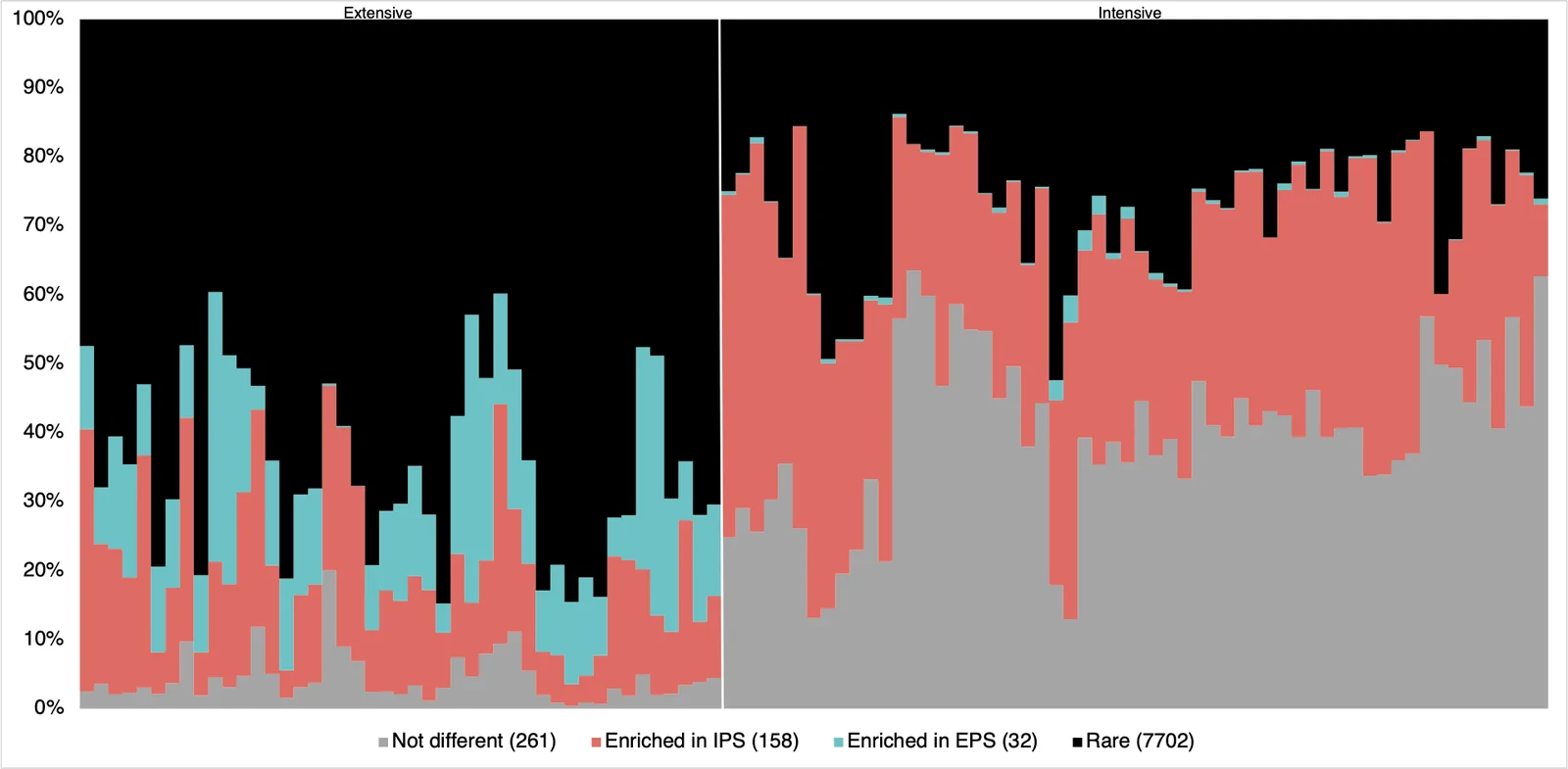
Simplified taxa plots showing the relative abundance of ASVs in the cecal contents of 35-day-old broilers from extensive or intensive production systems. ASVs that were found to be enriched in the microbiota of extensively or intensively raised birds are shown in green and red, respectively. ASVs that were not differently abundant between the systems are shown in gray. ASVs observed in less than 20 birds were aggregated into a single category called rare, and their relative abundance is indicated in black. The numbers between brackets indicated how many ASVs were included in each grouping category.

Differential abundance analyses at the ASV level were performed after aggregating all ASVs not seen in more than 20 birds into a single feature named “rare.” This reduced the number of ASVs from 8,153 to 452 ASVs that represented an average relative abundance of 72.6% ± 9.5 (mean ± SD) of the microbial community in IPS birds and 35.5% ± 9.5 of the community in EPS. Within these 452 ASVs, 158 and 33 ASVs were enriched in broilers from IPS and EPS, respectively. ASVs that were aggregated as rare were enriched and composed most of the microbial community (64.5% ± 9.5) in EPS birds. The microbiota of IPS birds was mostly composed of ASVs that were not differentially abundant between the systems (39.8% ± 12.3). It was also noteworthy that ASVs that were enriched in IPS birds were consistently found in EPS birds, with an average relative abundance reaching 16.5% ± 9.0 in the latter, whereas the ASVs enriched in EPS birds rarely occurred in IPS broilers, which had an average relative abundance of only 0.6% ± 0.8 in the latter (Fig. 3; Table S1). Without performing aggregation of rare ASVs, differential abundance analyses resulted in 249 ASVs enriched in EPS broilers and 130 ASVs enriched in IPS broilers.

### The family Enterobacteriaceae is enriched in IPS microbiotas

Differential abundance analyses at the family level were performed after combining families not detected in more than 20 birds into a single category called rare, which reduced the number of bacterial families from 97 to 53. Analyses of these 53 families indicated that five bacterial families were unique to the microbiota of EPS broilers, namely, Deferribacteraceae (0.8% ± 1.0, *P* < 0.001), Elusimicrobiaceae (0.9% ± 3.4, *P* < 0.001), Spirochaetaceae (2.7% ± 4.0, *P* < 0.001), Synergistaceae (0.4% ± 0.5, *P* < 0.001), and *Victivalles vadin BE97* (0.1% ± 0.2, *P* < 0.001). In addition, nine families were found to be enriched in EPS birds, whereas seven families were enriched in IPS birds, including Enterobacteriaceae (*P* < 0.001). The average relative abundance of Enterobacteriaceae in IPS and EPS broilers was 2.4% ± 3.9 and 0.2% ± 0.3, respectively (see Fig. S5).

### IPS cecal microbiotas are missing microbes

At the taxonomic level, 49.4% of the total number of taxa was shared between the cecal microbiota of broilers from both systems, whereas 41.5% and 9.1% of taxa were unique to the cecal microbiota of broilers raised in EPS and IPS, respectively. Analyses of features assigned to the genus and species levels indicated 10 genera and 6 species to be missing from the microbiota of EPS broilers, whereas 55 genera and 31 species were indicated to be missing from the microbiota of IPS broilers (Table S2). All taxa found to be missing from EPS broilers presented rare occurrences in IPS broilers, and the same was true for most of the taxa found to be missing from IPS birds. However, some taxa commonly found in EPS birds were completely absent from IPS birds, namely, the species *Bacteroides plebeius* [2.3% ± 3.9 (mean relative abundance ± SD) , *P* < 0.001], *Bacteroides salanitronis* (0.2% ± 0.4, *P* < 0.001), as well as the genera *Alloprevotella* (2.2% ± 2.9, *P* < 0.001), Prevotellaceae UCG-001 (1.6% ± 0.8), *Mucispirillum* (0.8% ± 1.1, *P* < 0.001), *Elusimicrobium* (0.9% ± 3.4, *P* < 0.001), and Synergistetes (0.4% ± 0.5, *P* < 0.001).

**FIG 4:**
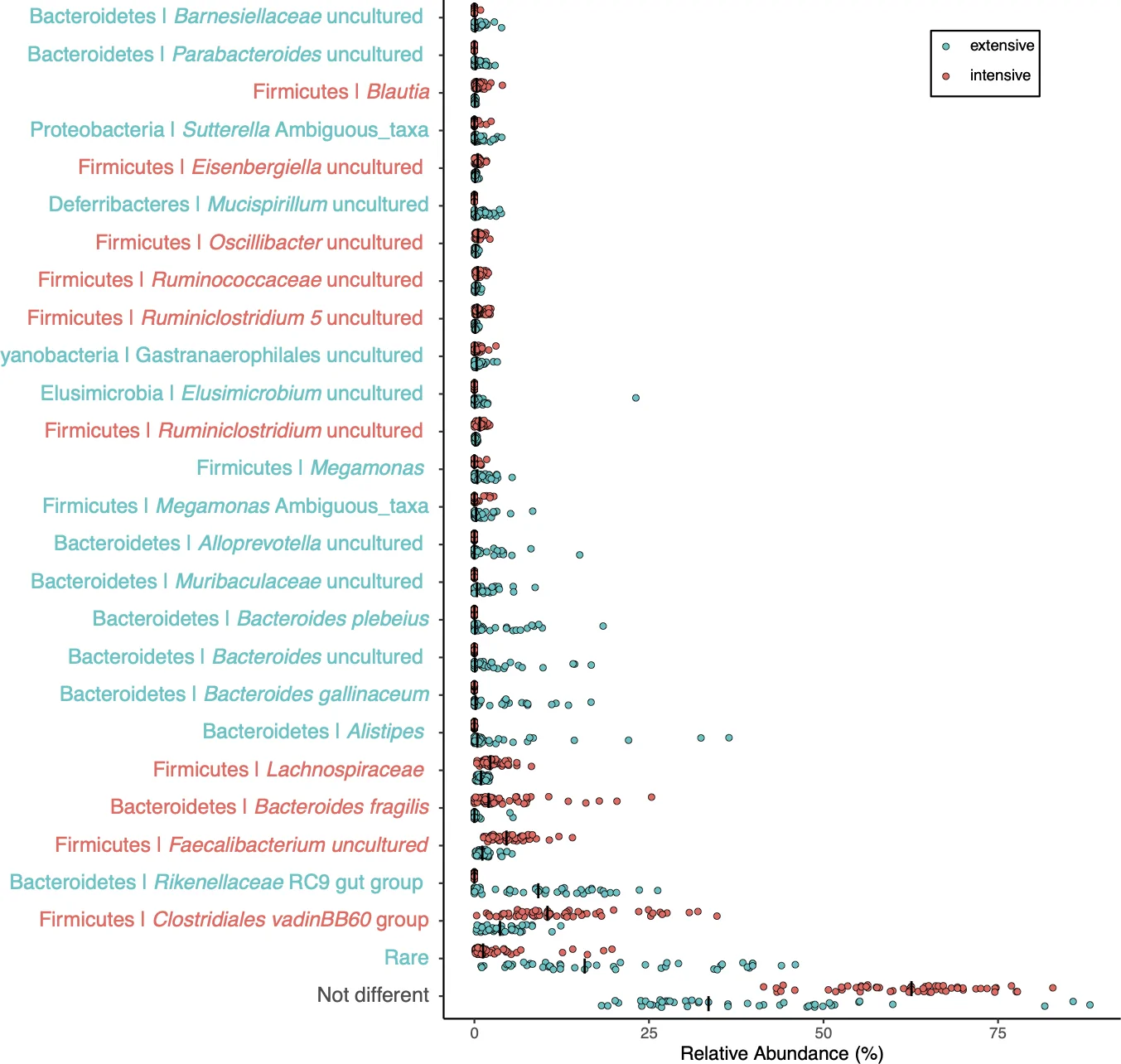
The relative abundance of microbial taxa that were shown to be differently abundant in the cecal microbiota of 35-day-old broilers from extensive or intensive production systems. Taxa names are color coded according to the system in which they were found to be enriched: green for an extensive system and red for an intensive system. Dots represent the relative abundance of taxa in individual samples. Taxa observed in less than 20 birds were combined into a single category called rare and shown to be significantly enriched in EPS broilers. The relative abundance of taxa that were not differently abundant between the systems is shown as “not different.”

**FIG 5:**
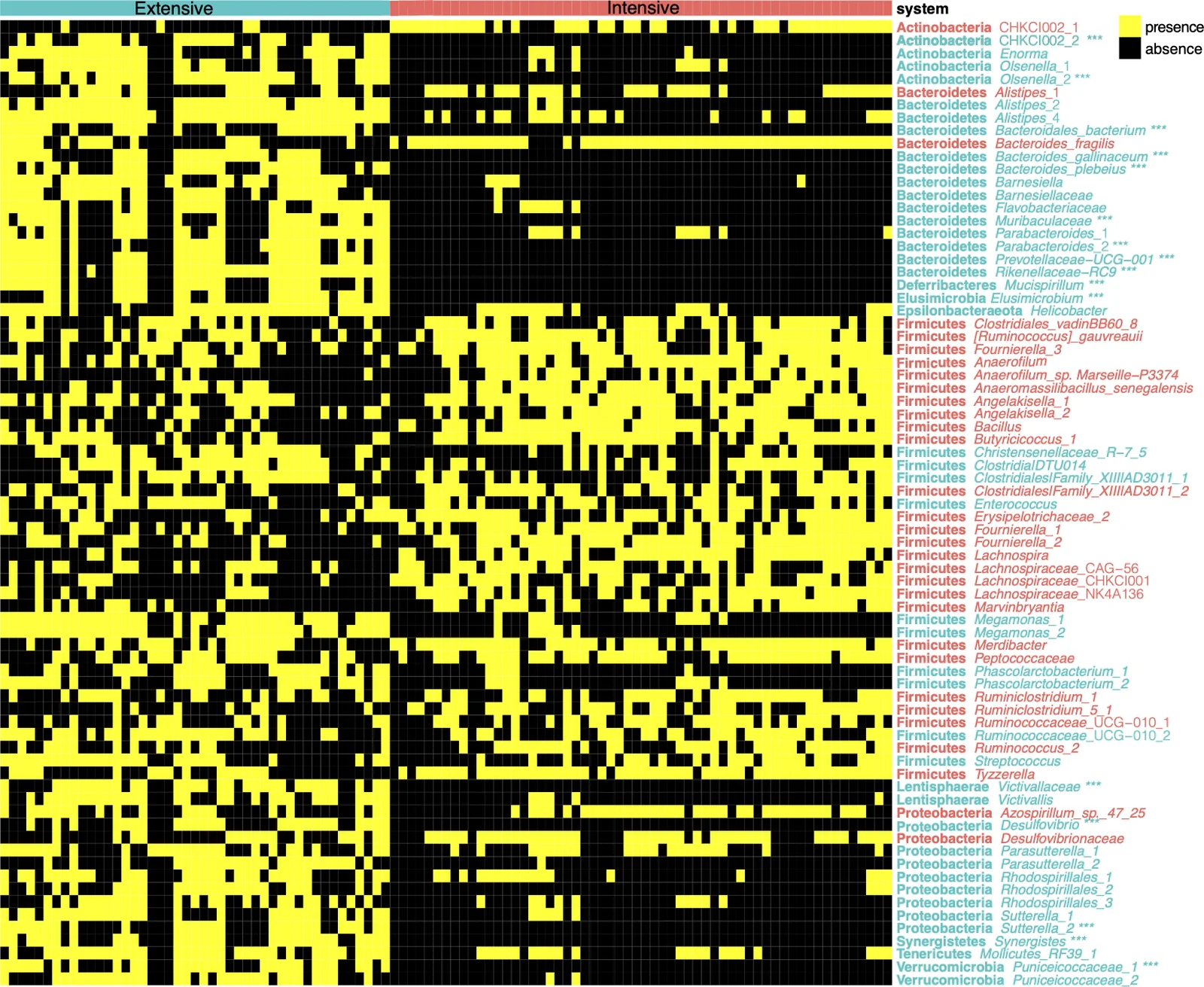
Heatmap indicating the presence (yellow) and absence (black) of core microbes in the microbiota of IPS and EPS broilers. Each column represents a sample and is colored green (left side) or red (right side) if obtained from EPS or IPS broilers, respectively. Taxa names are colored green or red if found to be core taxa within the microbiota of EPS or IPS broilers, respectively.

Differential abundance analyses at the taxa level were performed after combining all taxa present in less than 20 birds as a single taxon called rare, which reduced the number of taxa from 692 to 241. We found 39 taxa enriched in EPS birds and 36 taxa enriched in IPS birds. Most of the differentially abundant taxa showed an average relative abundance below 0.5% (Fig. 4; Fig. S6). A total of 164 taxa were assigned as core components of the broiler cecal microbiota, 90 of which were shared between birds in both systems (Fig. S7). In addition, 44 taxa were assigned as core in EPS birds, and 31 taxa were assigned as core in IPS birds (Fig. 5). The cecal microbiota of IPS broilers was shown to be depleted of taxa that were core members of EPS birds, such as *Olsenella* (0.3% ± 0.22 vs 0.01% ± 0.03, *P* = 0.001), Bacteroidales (38.1% ± 3.0 vs 20.7% ± 1.9, *P* < 0.001), *Bacteroides gallinaceum* (2.6% ± 4.2 vs 0%, *P* < 0.001), *Bacteroides plebeius* (2.3% ± 3.9 vs 0%, *P* < 0.001), Muribaculaceae (1.3% ± 1.9 vs 0%, *P* < 0.001), *Parabacteroides* (0.9% ± 0.7 vs 0.4% ± 0.9, *P* < 0.001), Prevotellaceae UCG-001 (1.6% ± 0.8 vs 0%, *P* < 0.001), Rikenellaceae RC9 (9.2% ± 7.6 vs 0%, *P* < 0.001), *Mucispirillum* (0.8% ± 1.1 vs 0%, *P* < 0.001), *Elusimicrobium* (0.9% ± 3.4 vs 0%, *P* < 0.001), Victivallaceae (0.1% ± 0.2 vs 0%, *P* < 0.001), *Desulfovibrio* (0.4% ± 0.5 vs 0.0 ± 0.1%, *P* < 0.001), *Sutterella* (0.9% ± 0.8 vs 0.1% ± 0.4, *P* < 0.001), Synergistetes (0.4% ± 0.5 vs 0%, *P* < 0.001), and Puniceicoccaceae (0.2% ± 0.2 vs 0%, *P* < 0.001). The microbiota of EPS birds presented a lower frequency of Firmicutes that were core members of IPS birds, although complete depletion was not observed for any taxa in EPS birds.

### The EPS microbiota has greater predicted functional potential

**FIG 6:**
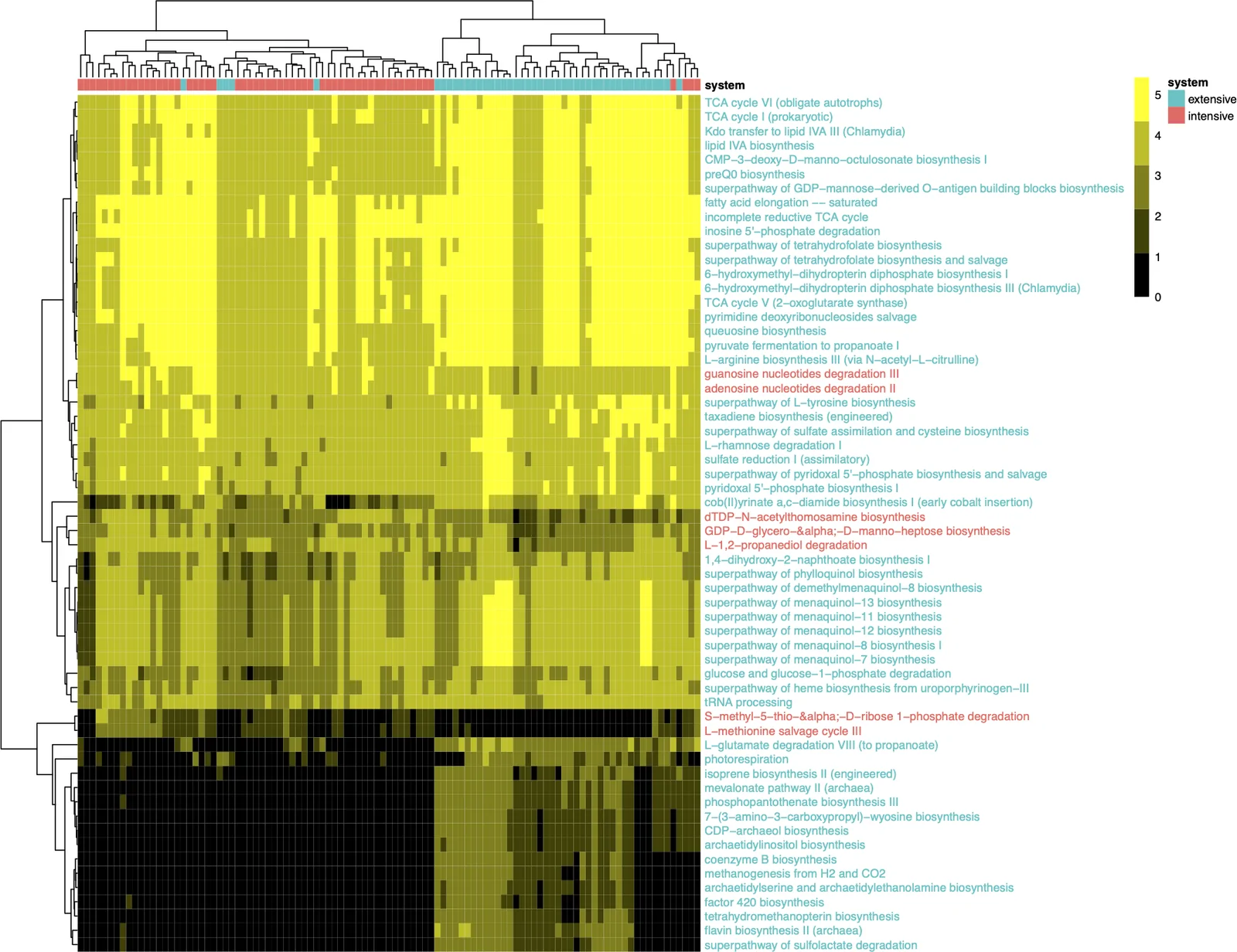
Heatmap indicating the log-transformed abundance of predicted pathways shown to be differently present in the microbiota of broilers from extensive (green) and intensive (red) rearing systems. Each column represents a sample and is colored green or red if obtained from EPS or IPS broilers, respectively. Pathway names are colored green or red if found to be enriched in the microbiota of EPS or IPS broilers, respectively.

Principal component analysis of predicted Enzyme Commission genes and MetaCyc pathways indicated a clear separation between samples obtained from EPS and IPS broilers (Fig. S8). A total of 167 pathways were shown to be differentially present, of which 95 were enriched in EPS broilers and 52 were enriched in IPS broilers. With an effect size of 0.5 as a threshold, 60 pathways were identified as biologically relevant (Fig. 6). A total of 75 ASVs were estimated to contribute to the enriched pathways, and most of the contributing ASVs were assigned to the order Clostridiales (Table S3). The microbiota of EPS broilers presented 53 enriched pathways that were mainly involved in the biosynthesis of amino acids (L-arginine, L-serine, and L-tyrosine), cofactors and vitamins (B6, B9, B12, K2, coenzyme A), fatty acids, and carbohydrates. In addition, the microbiota of EPS broilers showed several enriched pathways related to nutrient degradation and assimilation, including rhamnose degradation and sulfur assimilation, and pathways related to the generation of precursor metabolites such as methane and propionic acid. On the other hand, only seven pathways were shown to be enriched in the microbiota of IPS broilers. These pathways were shown to be involved in the biosynthesis of L-methionine, dTDP-N-acetylthomosamine (an important antigen in the outer membrane of Enterobacteriaceae), and heptose sugars (commonly found on the cell surface of many bacteria).

### Isolation of chicken commensals and whole-genome sequencing

**FIG 7:**
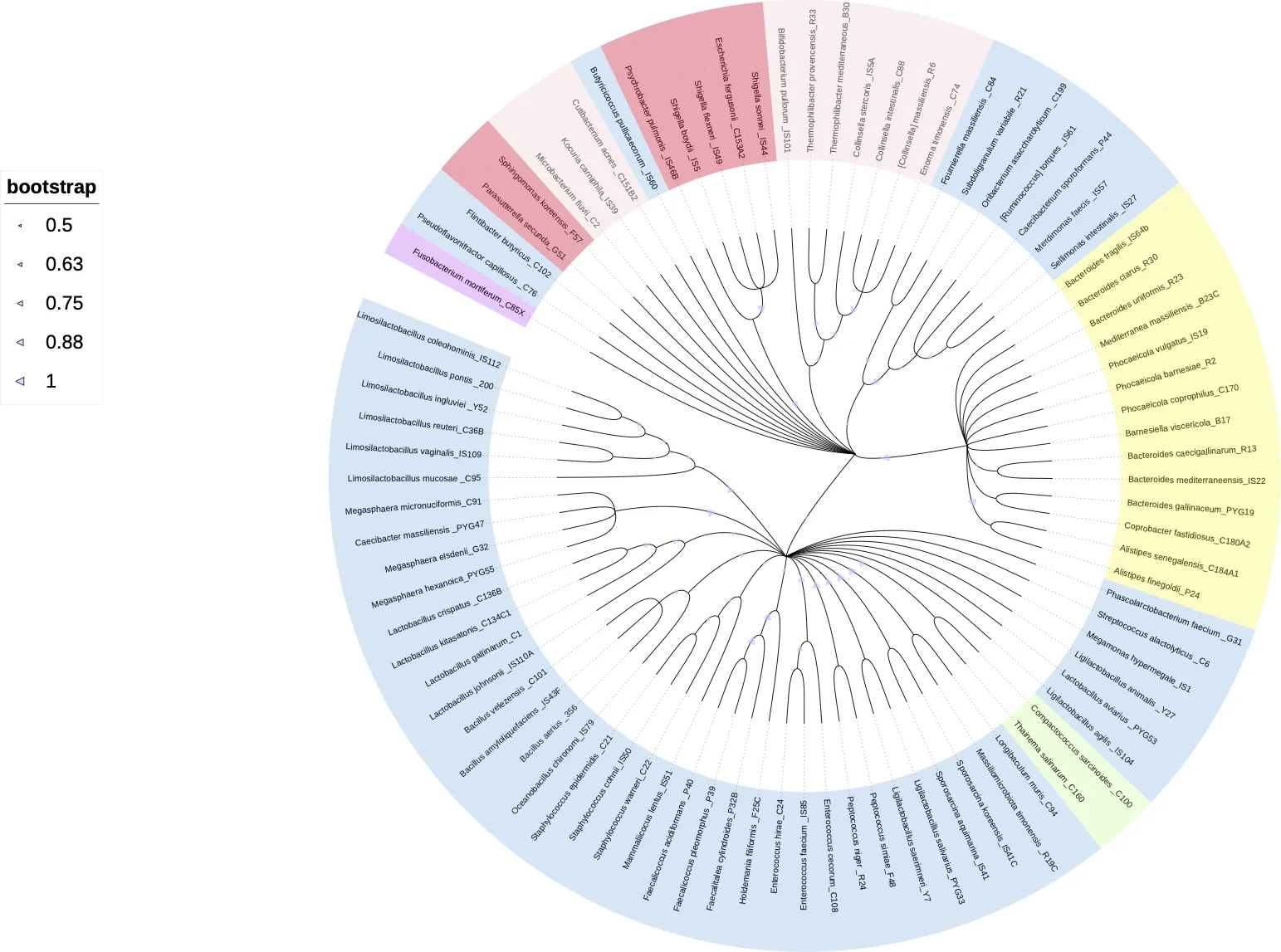
Phylogram showing the diversity of the 87 bacterial species isolated from the cecal microbiota of chickens. Letters and numbers after the species name indicate the identification number of a representative isolate for each species. The colors in the circle represent the phyla Actinobacteria (salmon), Bacteroidetes (yellow), Cyanobacteria (green), Firmicutes (blue), Fusobacteria (purple), and Proteobacteria (pink).

**FIG 8:**
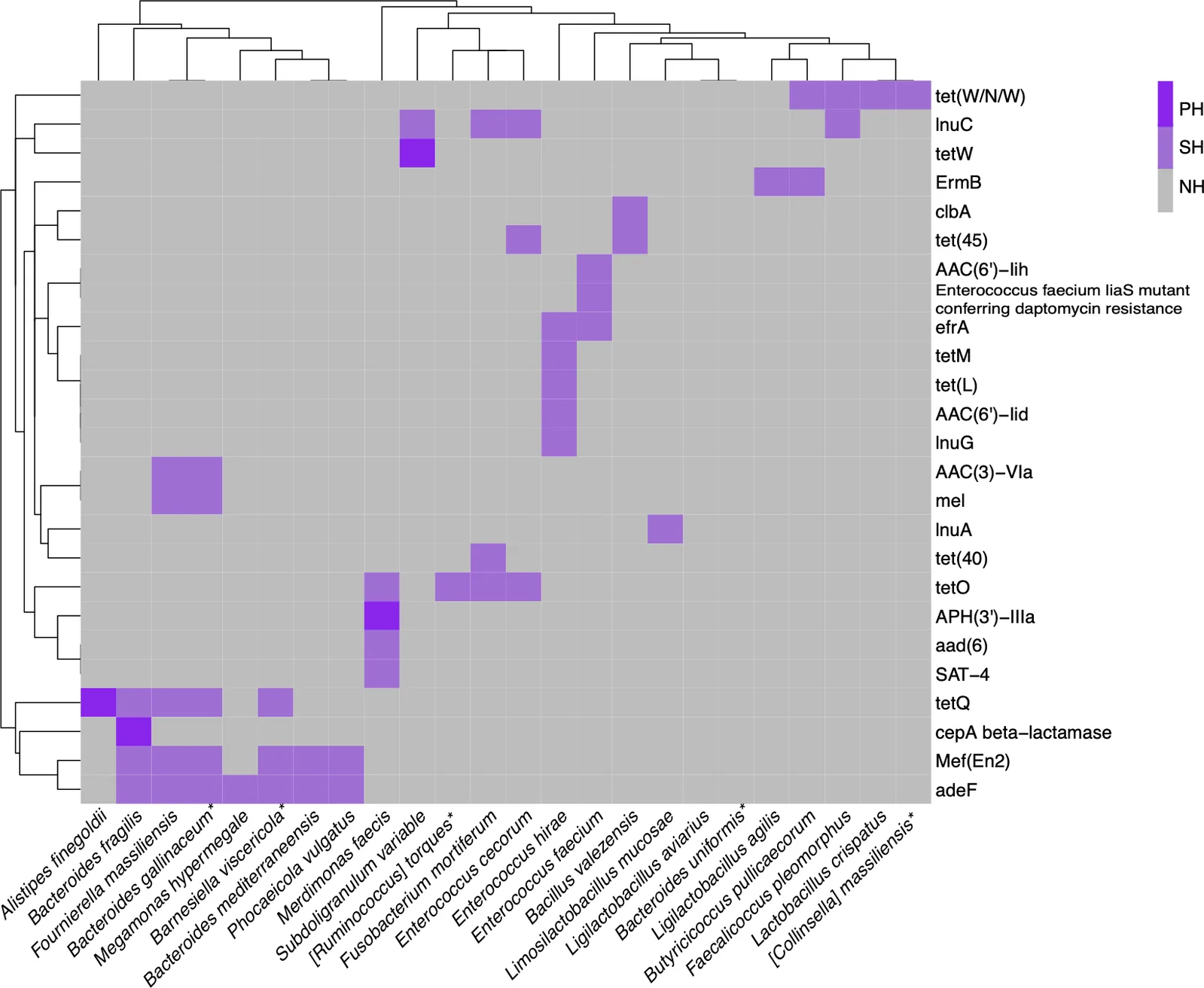
Heatmap indicating the presence of genes conferring antimicrobial resistance in the genomes of selected isolates. The dark purple color indicates a resistance gene identifier (RGI) match 100% identical to the reference protein sequence (perfect hit, PH); the light purple color indicates an RGI match with a bit-score greater than the curated BLASTP bit-score cut-off (strict hit, SH); and the gray color indicates no RGI match (NH). Species names followed by an * indicate isolates considered novel species.

We collected and identified 410 isolates, which were assigned to 87 species from 6 phyla. Most isolates were members of Firmicutes, comprising 53 species, followed by 14 species from Bacteroidetes, 10 from Actinobacteria, 7 from Proteobacteria, 2 from Cyanobacteria, and 1 species from Fusobacteria (Fig. 7; Table S4). We selected 24 isolates from the phyla Actinobacteria, Bacteroidetes, and Firmicutes for whole-genome sequencing (WGS) analysis, of which 5 were considered new species. The isolates considered as new species had low OrthoANI values compared to reference genome sequences of *Collinsella massiliensis* (75.93%), *Bacteroides gallinaceum* (74.2%), *Bacteroides uniformis* (75.03%), *Barnesiella viscericola* (85.73%), and *Ruminococcus torques* (74.3%) (Table S5). The presence of genes encoding bacteriocins and identified virulence factors is indicated in Tables S6 and S7. The presence of antibiotic resistance genes is illustrated in Fig. 8.

## DISCUSSION

Identifying bacteria that are normal inhabitants of the cecal microbiota of broilers is an initial step in using microbial manipulation in favor of broiler production. As intensive production practices may hinder the transmission of commensal microbes across generations (29), exploring potential missing microbes in intensively raised broilers compared with broilers reared in extensive systems can guide the development of microbial strategies to manipulate broiler health and performance. In the current study, we characterized the cecal microbiota of market-aged broilers raised in 22 independent commercial farms under intensive and extensive production systems. We found that the microbiota of EPS broilers was significantly different and had higher phylogenetic diversity than the microbiota of IPS broilers. Numerous bacteria were completely absent from IPS broilers compared with EPS broilers. Among these missing bacteria, some were cored in broilers from EPS, including *Olsenella*, *Bacteroides gallinaceum*, *Bacteroides plebeius*, *Parabacteroides*, *Mucispirillum*, *Elusimicrobium*, Victivallaceae, *Sutterella*, *Desulfovibrio*, and Synergistetes. On the other hand, a few missing bacteria, such as Fusobacteria and Patescibacteria, were only occasionally observed in EPS broilers and therefore considered less relevant.

At the phylum level, there was a clear difference between the systems, with the cecal microbiota of EPS broilers presenting a higher relative abundance of Bacteroidetes and Proteobacteria, while the cecal microbiota of IPS broilers was dominated by Firmicutes. This was consistent with previous studies comparing poultry in extensive and intensive systems (25, 27, 30 – 33) and has also been observed in humans living in rural and urbanized societies (34, 35), as well as in wild and captive rodents (22, 36). The similarity among results is not surprising since broilers are captive animals and current production practices resemble the changes in lifestyle that human society underwent during urbanization, which include sanitation, the use of antibiotics, and reduced microbiota transmission between mothers and offspring (37).

Previous studies have indicated that spore-forming and aerotolerant bacteria, such as most Firmicutes and Proteobacteria, are likely acquired from the environment, whereas bacteria not equipped to survive in aerobic conditions, such as Bacteroidetes (non-spore-forming strict anaerobes), need to be transmitted from mother to offspring (38). EPS broilers are more likely to present higher Bacteroidetes abundance due to their contact with a less sanitized environment and potential encounter with fecal material from mature birds, while IPS broilers are more likely to be colonized by Firmicutes present in the environment. Non-spore-forming strict anaerobes present a higher colonization capacity and a higher degree of host adaptation than that of spore-formers and aerotolerant bacteria (12). In that way, the reduction of Bacteroidetes and other obligate anaerobes such as *Megamonas* and *Parasutterella* indicates that intensively raised broilers are missing host-adapted species that likely co-evolved with birds in nature.

Among enriched Bacteroidetes species in the cecal microbiota of EPS broilers, species *Alistipes*, *Barnesiella*, *and Parabacteroides* have been shown to be efficient colonizers of the broiler ceca (39, 40) and to be enriched in chicks raised in contact with an adult hen (41). Specifically, *Alistipes*, one of the first colonizers of the chicken GIT (42), has been positively correlated with broiler body weight (43) and has been shown to be more abundant in the ceca of healthy broilers compared to coccidiosis-infected broilers (33) and in the ceca of free-range chickens compared to commercial broilers (28). *Barnesiella* has been shown to be enriched in the cecal material of free-range chickens (28), high-performing broilers (33), and chicks colonized with cecal material (40). *Parabacteroides* has been shown to be positively correlated with broiler body weight (44) and to be more abundant in older chickens (28, 45 – 47), which could be an indicator of a more mature microbiota.

Within the genus *Bacteroides*, *Bacteroides thetaiotaomicron* and *Bacteroides fragilis* were enriched in the microbiota of IPS broilers, whereas *Bacteroides salinitronis* DSM 18170, *Bacteroides plebeius*, and *Bacteroides gallinaceum* were enriched in the cecal microbiota of EPS broilers. *B. plebeius*, *B. gallinaceum*, and *B. salinitronis* have been previously shown to be chicken-adapted species, whereas *B. thetaiotaomicron* has been shown to be human adapted (48), thus indicating that IPS broilers are likely being colonized by *Bacteroides* of human origin instead of chicken commensals. The reduction of host-adapted species may affect several aspects of bird physiology (6, 9, 49). For example, it was recently shown that week-old broilers with high *Bacteroides* abundance had increased short-chain fatty acid concentration, higher cecal claudin-1 and IL-10 expression, and lower expression of interleukin-1β compared to broilers with low *Bacteroides* abundance, suggesting that *Bacteroides* can promote polysaccharide degradation, improve intestinal barrier, and modulate immune responses toward downregulation of inflammatory pathways (50).

Within the phylum Firmicutes, *Megamonas*, a core member of the broiler cecal microbiota, was shown to be less abundant in IPS broilers. *Megamonas* has been shown to be an efficient colonizer of the broiler gut and to be enriched in broilers inoculated with adult cecal content and defined communities (6, 39, 41, 51, 52). Interestingly, enriched *Megamonas* has also been observed in wild and free-range birds compared to birds in captivity (26, 28, 53, 54). In addition to being a free hydrogen utilizer and short-chain fatty acid producer, *Megamonas* has been shown to inhibit *Salmonella* growth *in vitro* (55, 56), which warrants further exploration of the function of this genus in the chicken GIT.

Within the phylum Proteobacteria, the cecal microbiota of EPS broilers showed a higher relative abundance of *Sutterella*, *Parasutterella*, and *Desulfovibrio*. *Desulfovibrio* is an effective colonizer of the chicken ceca (39, 41), which can consume free hydrogen present in the gut environment (42). *Parasutterella* has been identified as a core member of the gut microbiota of 35-day-old broilers (46), and *Sutterella* has been positively associated with broiler body weight (44). Although there has been no specific study evaluating the role of these genera in the gut microbiota of chickens, *Parasutterella* has been shown to significantly impact host physiology by modulating bile acid and cholesterol metabolism in mice (13).

In the current study, six phyla were exclusively detected in extensively raised birds. Among these six unique phyla, Fusobacteria was a minor component of the broiler microbiota, which agrees with other studies (42). Despite its relative low abundance (0.1% ± 1.3), some Fusobacteria species can degrade uric acid (1, 55), which could be of importance to broiler physiology. Spirochaetes were detected in 46% of the EPS broilers, with an average relative abundance of 2.7% ± 4.1. Members of this phylum have been reported to be enriched in free-range chickens (57) and Indian native chicken breeds (32) compared to commercial broilers. The main Spirochaetes detected in our study were *Treponema*, *Sphaerochaeta*, and *Brachyspira*, which have been considered as potential pathogens (58). Some *Treponema* species can degrade cellulose and xylan (59), and the observed enrichment in EPS chickens could result from access to high-fiber substrates. The phylum Elusimicrobia was present in 50% of the EPS broilers, with an average relative abundance of 0.9% ± 3.4. *Elusimicrobium* was previously shown to be missing from commercial broilers compared to indigenous breeds (32) and to be a core microbe in the cecal microbiota of 81-day-old free-range chickens (28). Interestingly, an increase in *Elusimicrobium* was observed in the microbiota of laying hens fed with insect-based diet (60). As a common member found in the GIT of insects (61), the presence of *Elusimicrobium* in EPS broilers is likely a consequence of these birds having access to insects as food sources. The phylum Synergistetes was detected in most EPS birds, with an average relative abundance of 0.4% ± 0.5. This phylum has been reported both as a minor and major component of the chicken gut microbiota (41, 62 – 65). Synergistetes species can degrade toxic compounds from plants (66) and were shown to be abundant in the ceca of wild capercaillie but absent from captive birds. Capercaillie relies on conifer plants as a feed source during the winter, and it is speculated that the low survival rates observed in captive birds re-introduced to wild environments result from a lack of Synergistetes within the gut microbiota of captive birds (53). The reduction in Synergistetes seen in captive birds, coupled with the impaired host ability to detoxify toxic compounds and digest plant materials, warrants further investigation of the impact of Synergistetes on the cecal microbiota of broilers.

The microbiota of IPS broilers had a reduced frequency of *Olsenella* and *Victivallis*. In contrast with our results, a previous study observed *Olsenella* to be increased in indoor hens compared to outdoor-reared hens (25). It is possible that the contradictory results are due to differences in *Olsenella* species present across studies. In our study, the only ASV assigned to the species level was *Olsenella* sp. Marseille-P3256, which has recently been reclassified as *Thermophilibacter mediterraneus* (67). We were able to isolate *Thermophilibacter mediterraneus* and *Thermophilibacter provencensis*, which are former *Olsenella* species that could be employed in future studies to evaluate the impact of *Olsenella* on host physiology. In addition, in agreement with our results, *Victivallis* was previously shown to be enriched in extensively raised chickens (25, 28), which could be related to access to insects, as *Victivallis* was shown to be enriched in chickens fed insect larvae (68).

The microbiota of IPS broilers showed an enrichment in *Blautia*, *Faecalibacterium*, and *Oscillibacter*, which were core microbes shared by birds in both systems. *Blautia* and *Oscillibacter* were previously observed to be enriched in indoor- compared to outdoor-raised chickens (25). Interestingly, chicks inoculated with a competitive exclusion product containing *Blautia* and *Oscillibacter* were shown to have a lower relative abundance of *Blautia* than non-inoculated chicks, while no *Oscillibacter* was detected in either group (69). This suggests that *Blautia* and *Oscillibacter*, although ubiquitous in the broiler cecal microbial community, can be displaced by other bacteria if other bacteria are available. *Faecalibacterium* is a major butyrate producer (70) that has been associated with improved growth performance and gut health in broilers (21, 33, 44, 71 – 73); however, the characterization of the effect of *Faecalibacterium* on broiler physiology is still needed. In addition, several genera with a relative abundance lower than 0.5% were shown to be enriched in the microbiota of IPS broilers. Among these enriched genera, *Bacillus* and *Butyricicoccus* have been previously mentioned as core members of the broiler microbiota (28, 31, 74). Several *Bacillus* species have been used as probiotics and suggested to confer benefits to broilers (75); however, *Bacillus* species are usually not effective colonizers of the chicken gut and need to be constantly provided to exert effects (12, 41). *Butyricoccus* has been considered a potential probiotic due to its production of butyrate (76) and positive association with broiler performance and disease resilience (72, 77); however, it has been reported that pure cultures of *Butyricicoccus pullicaecorum* inoculated in day-old chicks failed to colonize the ceca (39).

A greater predictive functional potential observed in the microbiota of EPS broilers may be related to different dietary patterns between systems. Although greater functional potential could be considered beneficial, the pathways enriched in the EPS microbiota may not necessarily benefit IPS broilers, which are fed relatively simplified diets. Nonetheless, identifying and harvesting bacteria capable of providing functional potential could aid the use of alternative feed ingredients if coupled with the supplementation of specific bacteria that can utilize these ingredients. Moreover, bacteria harvested from EPS chickens could potentially have a lower incidence of antimicrobial resistance genes and a higher ability to inhibit pathogen growth. A limitation of our results is that the accuracy of functional predictions based on 16S rRNA sequencing is dependent upon the availability of reference genomes, which could potentially result in limited annotation of particular ASVs. Future studies using techniques such as metagenomic sequencing will help improve the accuracy of the functional annotation of microbial communities in EPS and IPS chickens.

### Conclusion

In the current study, we focused on bacteria from EPS chickens since these bacteria are more likely to be host adapted and to have evolved with chickens in nature. We identified *Olsenella*, *Alistipes*, *Bacteroides*, *Barnesiella*, *Parabacteroides*, *Megamonas*, and *Parasutterella* as core microbes within the broiler cecal microbiota to be further investigated for their effects on bird physiology and potential applications as next-generation probiotics. These genera seem to be depleted in IPS broilers but are frequently found in EPS broilers and readily colonize the ceca after a single exposure. The collection of bacterial isolates generated in this study will be used as a resource to further explore how differences in microbiota composition can influence bird physiology and to elucidate the role of individual species within the microbial community.

## MATERIALS AND METHODS

### Samples

Farms that participated in this research project were recruited with the assistance of poultry industry workshops, producer associations, and local veterinarians. Details about the research project were introduced to participating producers, and samples were collected with research consent. Cecal samples from IPS broilers (*n* = 59) were collected from 12 independent commercial farms. The broilers were euthanized on farm using cervical dislocation, and the cecal contents were collected using sterile techniques into an empty tube or a tube containing liquid casein yeast (LCY) media supplemented with 30% glycerol and 0.05% L-cysteine. Samples were transported on dry ice and stored at −80°C until use. Cecal samples from EPS broilers (*n* = 46) were collected from 10 independent commercial small-scale farms that supply poultry products to local farmers’ markets. Among these 10 farms, one farm that raised free-range broilers in an organic system was visited, and samples were collected as described for IPS broilers. Samples from the remaining nine EPS farms were collected from a provincially inspected slaughterhouse. Specifically, broilers were electrically stunned, bled, and eviscerated, and intestinal tracts (from ileum to cloaca) were collected in sterile plastic bags and transported on ice to a laboratory within 3 h. Cecal tissues were subsequently dissected in an anaerobic chamber (Bactron 300, Sheldon Manufacturing Incorporated; gas conditions: 5% CO_2_, 5% H_2_, and 90% N_2_) to collect the cecal contents as described above.

Cecal samples from additional chickens were collected in LCY supplemented with 30% glycerol and 0.05% L-cysteine for culturing and isolating bacteria. Samples were obtained from 2-year-old backyard bantam rosters (*n* = 2), 17-week-old roosters from heritage breeds raised without antibiotics and with access to the outdoors (*n* = 5), 1-, 5-, and 40-week-old layers raised in an organic system with access to the outdoors (*n* = 5 per age), and 40-week-old layers raised in cages in an intensive system (*n* = 5). These birds were euthanized on farm using cervical dislocation, and the cecal contents were transported on dry ice and stored at −80°C until use.

### DNA extraction

The extraction of DNA from cecal contents, reagent control, and a gut microbial community standard (ZymoBIOMICS, ZymoResearch) were performed using the QIAamp DNA Stool Mini Kit (Qiagen Inc., US) according to the manufacturer’s instructions with the addition of a bead-beating step. Approximately 100 mg of cecal content was mixed with Inhibitex buffer and 2.0 mm garnet beads (BioSpec Products, Bartlesville, OK, USA) and lysed by bead-beating twice at 6.0 m/s for 30 s (FastPrep-24TM 5G, MP Biomedicals). The purity and concentration of the extracted DNA were assessed using a Nanodrop 2000 spectrophotometer (Thermo Scientific) and a Quant-iT PicoGreen dsDNA Assay Kit (Thermo Scientific). The Illumina 16S Metagenomic Sequencing Library Preparation Protocol targets the V3-V4 region of the 16S rRNA gene (primers forward 5′ TCGTCGGCAGCGTCAGATGTGTATAAGAGACAGCCTACGGGNGGCWGCAG and reverse: 5′ GTCTCGTGGGCTCGGAGATGTGTATAAGAGACAGGACTACHVGGGTATCTAATCC). Each 25 µL of polymerase chain reaction (PCR) reaction contained 12.5 µL of 2× KAPA HiFi HotStart ReadyMix, 5 µL of 1 µM forward primer, 5 µL of 1 µM reverse primer, and 2.5 µL of DNA template (5 ng/µL). The PCR program consisted of an initial denaturation step of 3 min at 95°C, followed by 25 cycles of 95°C for 30 s, 55°C for 30 s, 72°C for 30 s, and a final extension step of 72°C for 5 min. Amplicons were purified using AMPure XP beads prior to and after the attachment of Illumina sequencing adapters. The final library was diluted to 4 nM and sequenced using paired-end 2 × 300 cycles on an Illumina MiSeq Platform (Illumina Inc., San Diego, CA, USA). All DNA extractions and sequencing procedures were performed by the same person.

### 16S rRNA amplicon sequencing analysis

Raw sequencing data were processed using Quantitative Insight into Microbial Ecology 2 (QIIME 2 v2021.4) (78) and DADA2 for pairing, denoising, de-replication, and chimera filtering (79). Sequences were truncated at 270 (forward) and 220 (reverse) base pairs based on the median quality score and discarded if they presented more than six expected errors. Mafft and fastree methods (80, 81) were used to align sequences and generate phylogenetic trees. Taxonomy was assigned using the q-2-feature-classifier plugin (82). The Naïve Bayes classifier (83) was pretrained on the SILVA 138 QIIME compatible database (84). Sequences were clustered at 99% identity using majority taxonomy strings. Data were analyzed using phyloseq v.1.40.0 (85), microbiome v. 1.18.0 (86), and qiime2R v. 0.99.6 (87) packages in R v.1.4.1717 (88). ASVs assigned to the mitochondria family, chloroplast order, archaea kingdom, or unassigned were removed from the data set, and the remaining reads were rarefied at an even count for downstream analysis. Phylogenetic diversity, Chao1, and Simpson indices were used to evaluate alpha diversity. The Bray-Curtis distance matrix and principal coordinates analysis (PCoA) were used to evaluate beta diversity. Hierarchical clustering was performed based on the Bray-Curtis distance matrix and the single linkage (“friends-of-friends”) method (stats package). Testing for differentially abundant features was performed at the ASV, taxa, family, and phylum levels. Analysis at the taxonomic level was performed by merging all ASVs exhibiting the same taxonomy string using the *tax_glom* function (phyloseq package). Differential abundance analyses were done using the limma-voom tool v.3.52.4 (89, 90), DESeq2 (v.1.36.0) with apeglm for logarithmic fold change shrinkage and false discovery rate (FDR) correction (91, 92), and the non-parametric factorial Kruskal-Wallis sum-rank test from the linear discriminant analysis effect size algorithm (93). To reduce the occurrence of type I error, features were considered differentially abundant if differences were consistently detected by a combination of the three methods, with an alpha level of 0.05. The core microbiota for each system was defined as taxa present in at least 50% of birds in each group (94). Taxa concomitantly assigned as core in the microbiota of IPS and EPS broilers were defined as members of the core cecal microbiota. Figures were generated using the ggplot2 v.3.4.0 (95) and pheatmap v.1.0.12 (96) packages.

A phylogenetic investigation of communities by reconstruction of unobserved states (PICRUSt2, v.2.1.4-b) (97) was used to predict the functional potential of the microbiota based on ASVs. Sequences were aligned to reference trees using EPA-ng (98), and hidden state predictions were performed by Castor package v. 1.7.8 (99). Enzyme Commission numbers and MetaCyc pathways databases were used to predict microbial gene families and pathways.

### Bacterial isolation and identification

Cecal samples collected in LCY supplemented with 30% glycerol and 0.05% L-cysteine were thawed on ice, homogenized by vortexing, and serially diluted in 1× PBS inside an anaerobic chamber. Diluted samples were plated on nine types of media (Table S1) and incubated at 37°C using three gas conditions: aerobic, anaerobic (5% CO_2_, 5% H_2_, and 90% N_2_), and high CO_2_ (20% CO_2_, 10% H_2_, and 70% N_2_). After 72 h of incubation, single colonies with distinct morphologies were selected from each plate, streaked on fresh media plates, and incubated for 72 h to obtain isolates. The purified colonies were further characterized by Sanger sequencing. Amplicon PCR amplifying 16S rRNA gene was performed using primers 8F/926R (forward 5′-AGAGTTTGATCCTGGCTCAG-3′ and reverse 5′-CCGTCAATTCNTTTRAGT-3′). Each 50 µL of PCR reaction contained 5 µL of 10× Taq polymerase buffer (Invitrogen, Carlsbad, CA, USA), 2 µL of 10 µM forward primer, 2 µL of 10 µM reverse primer, 2 µL of 10 mM deoxynucleotide triphosphate mix (Invitrogen), 2 µL of 50 mM MgCl_2_ (Invitrogen), 0.5 µL of 1 U/µL Taq polymerase (Invitrogen), and 1 µL of nuclease-free water containing the harvested bacterial colony. The PCR program consisted of an initial denaturation step of 10 min at 94°C, followed by 40 cycles of 94°C for 30 s, 56°C for 30 s, 72°C for 1 min, and a final extension step of 72°C for 7 min. The amplicon products were purified using a GeneJET Gel Extraction and DNA Cleanup Micro Kit (Thermo Scientific). The purity and concentration of amplicon products were determined using Nanodrop 2000 (Thermo Scientific) and sent for Sanger sequencing (Molecular Biology Service Unit, University of Alberta). The taxonomy of the resultant sequences was analyzed using the rRNA/ITS database of 16S rRNA within the Basic Local Alignment Search Tool (BLAST) from the National Center for Biotechnology Information (NCBI). The maximum likelihood phylogenetic tree based on the 16S rRNA gene was constructed using MEGAX software, and the phylogenetic tree was visualized using Interactive Tree Of Life (iTOL v6) software. In addition, sterile 1× PBS was added to each type of plate, and the surface was scraped using an inoculation loop to collect microbial cells, which were mixed with LCY supplemented with 0.05% L-cysteine and 50% glycerol (final concentration, 25%), and stored at −80°C.

### WGS analysis

The extraction of genomic DNA from selected isolates was conducted using the Wizard Genomic DNA Purification Kit (Promega Corp., USA) following the manufacturer’s instructions. Purity and concentration of extracted DNA were determined using the Nanodrop 2000 spectrophotometer and the Quant-iT PicoGreen dsDNA Assay Kit (Thermo Scientific), respectively. The library preparation was performed using the NEBNext Ultra II DNA Library Prep kit (New England Biolabs Inc., CA, USA), followed by 150bp paired-end sequencing on an Illumina NovaSeq 6000 platform (Illumina Inc., USA). The quality of the resultant paired-end 150 bp sequences was analyzed using FastQC (v.0.11.9) (100), and sequence adapters were trimmed using Trimmomatic (v. 039) (101). Draft genome assemblies were performed using SPAdes assembler v.30.10.1 (102), and the quality of assembled genomes was evaluated using QUAST v.5.5.0 (103). For determination of taxonomy, the contig file of selected isolates was compared to reference genomes (RefSeq Genome Database) of suspected species using the OrthoANIu algorithm (104). Isolates that concomitantly presented 16S rRNA identity below 98% against the NCBI rRNA/ITS database using BLAST and OrthoANIu values lower than 95% against NCBI reference genomes were considered new species (105). Genome annotation was performed on the Rapid Annotation using Subsystem Technology system (106) and visualized using SEED Viewer (107). Genemark (108) and BLASTp (109) were used to predict genes and align the resulting amino acid sequences of coding genes. The amino acid sequences were aligned against the Bacteriocin database (110) for identification of genes encoding bacteriocins and the Virulence Factor Database for identification of genes encoding virulence factors using identity thresholds of >60% (Blastp). Additionally, the Comprehensive Antibiotic Resistance Database (111) was used to annotate antibiotic resistance genes using default parameters.

### Statistical analysis

Alpha-diversity indices were analyzed using one-way analysis of variance (ANOVA) and the Tukey honestly significant difference when data were normally distributed and the Kruskal-Wallis and Wilcoxon rank sum test with FDR adjustment if data were not normally distributed. Beta-diversity distance matrices were analyzed using PERMANOVA to test for differences in the distances to centroids and dispersion of the groups and permutation test for homogeneity of multivariate dispersions to test for homogeneity of dispersions between groups (vegan package). Differences in the gene abundance or MetaCyc pathways in extensive and intensive systems were identified using ANOVA-like differential expression (ALDEx) analysis (ALDEx2 package v.1.30.0 (112). An alpha level of 0.05 was considered for all the analyses.

## Data Availability

The data sets generated and analyzed in the current study are available in the NCBI SRA repository under accession no. PRJNA955389.

## SUPPLEMENTAL MATERIAL

Supplemental figures spectrum.02352-23-s0001.docx Fig. S1 to S8.

Supplemental tables spectrum.02352-23-s0002.xlsx Tables S1 to S7.

ASM does not own the copyrights to Supplemental Material that may be linked to, or accessed through, an article. The authors have granted ASM a non-exclusive, world-wide license to publish the Supplemental Material files. Please contact the corresponding author directly for reuse.
