# Supplementary material for: Investigating the cecal microbiota of broilers raised in extensive and intensive production systems
Source: Microbiol Spectr. 2023 Sep 27;11(5):e02352-23. doi: 10.1128/spectrum.02352-23 (PMC10581045; doi:10.1128/spectrum.02352-23)
Supplement: Supplemental figures — Fig. S1 to S8. [file spectrum.02352-23-s0001.docx]

**SUPPLEMENTAL FIGURES**

**Figure S1.** Shannon and Simpson indices indicating no differences in alpha-diversity between cecal samples obtained from 35-day-old broilers in extensive and intensive rearing systems.

**Figure S2.** Dendrogram showing hierarchical clustering of cecal samples from 35-day old broilers according to farm sources. Samples obtained from the same farm share the same color and identification. Identification of farms using extensive rearing practices are coded as “Farm_” followed by a number; whereas identification of farms using intensive rearing practices are coded as “Farm_” followed by a letter.

**Figure S3.** The relative abundance of bacterial phyla in the cecal microbiota of 35-day-old broilers in extensive (green) and intensive (red) rearing systems. Names in green and red indicate phyla that are more abundant in broilers in extensive and intensive rearing systems, respectively. Names in gray indicate no differences in phyla abundance between systems. Bold indicates phyla that are exclusively present in the microbiota of broilers from extensive systems.

**Figure S4. A**) The relative abundance of ASVs that are unique or shared in the cecal microbiota of 35-day-old broilers in extensive or intensive rearing systems, and the taxonomy at phylum-level of ASVs **B)** unique to each system and **C)** shared between the microbiota of birds in both systems.

**Figure S5.** The relative abundance of bacteria families in the cecal microbiota of 35-day-old broilers in extensive (green) and intensive (red) rearing systems. Names in green and red indicate families that are more abundant in broilers in extensive and intensive rearing systems, respectively. Bold indicates families that are exclusively present in the microbiota of broilers from extensive systems. The “not different” indicates the relative abundance of bacterial families that were not differently abundant between the systems.

**Figure S6.** The relative abundance of bacteria taxa with relative abundance lower than 0.5% in the cecal microbiota of 35-day-old broilers in extensive (green) and intensive (red) rearing systems. Names in green and red indicate taxa that are more abundant in broilers in extensive and intensive rearing systems, respectively.

**Figure S7.** Heatmap indicating the presence/absence of taxa that were found to be core within the microbiota of 35-day-old broilers reared in extensive and intensive systems. Each column represents a sample and is colored in green (left side) or red (right side) if obtained from EPS or IPS broilers, respectively.

**Figure S8.** Principal component analysis of PICRUST2-predicted **A)** Enzyme Commission genes and **B)** Metacyc pathways in the cecal microbiota of 35-day-old broilers reared in intensive and extensive rearing systems.
